# Polysaccharides from the Peel of *Hylocereus undatus* Promote Wound Healing by Reshaping the Skin Microbiome and Regulating Immune Balance

**DOI:** 10.3390/polym18111330

**Published:** 2026-05-28

**Authors:** Tao Zhou, Yunhua He, Ahluk Liew, Min Wang, Kit-Leong Cheong

**Affiliations:** 1 Guangdong Provincial Key Laboratory of Aquatic Product Processing and Safety, Guangdong Ocean University, Zhanjiang 524088, China; zhoutao732023@163.com (T.Z.); iheyunhua@gmail.com (Y.H.); 2 Guangdong Province Engineering Laboratory for Marine Biological Products, Guangdong Ocean University, Zhanjiang 524088, China; 3 Guangdong Provincial Engineering Technology Research Center of Seafood, Guangdong Ocean University, Zhanjiang 524088, China; 4 Guangdong Provincial Engineering Technology Research Center of Prefabricated Seafood Processing and Quality Control, Guangdong Ocean University, Zhanjiang 524088, China; 5 College of Food Science and Technology, Guangdong Ocean University, Zhanjiang 524088, China; 6 Guangdong Meichen Biotechnology Company Limited, Guangdong Suixi Dragon Fruit Science and Technology Small Courtyard, Zhanjiang 524088, China; ahlukliew@163.com; 7 College of Coastal Agriculture Sciences, Guangdong Ocean University, Zhanjiang 524088, China

**Keywords:** polysaccharides, skin wound, skin microbiota, immunomodulation, tissue regeneration

## Abstract

Polysaccharides isolated from the peel of *Hylocereus undatus* exhibit promising anti-inflammatory activity; however, the underlying mechanisms—particularly their modulatory effects on cutaneous microbiota composition and host immune responses—remain incompletely characterized. This study investigates the therapeutic potential of polysaccharides isolated from the peel of *Hylocereus undatus* in the management of inflammatory cutaneous wounds. The polysaccharide extracted from the peel of *Hylocereus undatus* via ultrasound-assisted extraction is an acidic heteropolysaccharide, with galacturonic acid and rhamnose as its dominant monosaccharide components. It exhibits low crystallinity, a porous structure, and good thermal stability. In a mouse wound model, treatment with the polysaccharide extracted from the peel of *Hylocereus undatus* significantly accelerated wound closure as early as day 3 (** *p* < 0.01). By day 9, the wound closure rate approached that of the positive control group and remained significantly higher than that of the untreated group (** *p* < 0.01), exceeding 90%. Treatment with the polysaccharide advanced the inflammatory peak, as evidenced by elevated anti-inflammatory cytokines (IL-10 and TGF-β) and suppression of the pro-inflammatory cytokine IL-6. Immunofluorescence staining confirmed that polysaccharide promoted cell proliferation and neovascularization at the wound site. In conclusion, polysaccharides isolated from the peel of *Hylocereus undatus* accelerate skin wound healing by modulating the skin microbiota, enhancing the anti-inflammatory response, and promoting tissue regeneration, highlighting its potential as a natural wound dressing.

## 1. Introduction

Skin wound healing is a complex process that relies on numerous cell types and mediators interacting in a highly intricate temporal sequence [1]. The repair process encompasses alterations in vascular permeability, initiation of inflammation, extracellular matrix (ECM) formation, angiogenesis, tissue remodeling, and ultimately scar tissue formation [2]. The healing of skin wounds is governed by a distinct and sophisticated functional mechanism [3]. Skin lesions have garnered considerable attention owing to their recurrence, prolonged course, and therapeutic challenges [4]. Severe skin damage can be life-threatening, and inflammation must be resolved before tissue remodeling and scar formation occur [5]. Against this backdrop, there is an urgent demand for minimally invasive, cost-effective, naturally derived wound dressings with substantial efficacy in promoting skin wound healing [6]. In recent years, beyond traditional cellular and molecular mechanisms, the microbial communities colonizing the skin surface have been identified to play a pivotal regulatory role in wound healing, providing a novel avenue for the development of innovative therapies.

The skin microbiome, also termed the skin microbiota, refers to the entire community of microorganisms naturally colonizing the human skin surface, together with the complex ecosystem they establish in association with the skin microenvironment [7]. Interactions between bacteria on the skin surface are critical to the pathophysiology of skin wounds and may contribute to delayed wound healing [8]. The skin microbiome directly modulates skin health and disease through interactions with various cells implicated in wound healing processes [9]. Notably, symbiotic microbiota interact with wound-repairing skin cells to promote skin barrier regeneration [10]. Potential crosstalk between the human microbiome and specific cell types involved in skin wound healing may enhance immune responses and help maintain skin barrier function [11]. Advancements in next-generation sequencing and bioinformatics approaches have greatly improved our understanding of the complex roles of microorganisms in skin wound healing [12]. Herein, we aim to elucidate the significance of these pathways in the pathophysiology of common inflammatory conditions, including wound healing. Therefore, modulating the wound healing process by intervening in the skin microbiome represents a highly promising strategy. Natural product-derived active polysaccharides, with their excellent biocompatibility and diverse biological activities, are regarded as ideal candidates for implementing this strategy.

The polysaccharides from the peel of *Hylocereus undatus* (HUPP) are a natural macromolecular polymer primarily extracted from the fruit peel. It is composed of numerous monosaccharide units linked together by glycosidic bonds [13,14]. The composition and content of peel polysaccharides vary with the dragon fruit variety, and they exhibit a range of biological activities, including antioxidant, anti-fatigue, antitumor, and antibacterial activities [15]. Although HUPP has been extensively studied over the years, its role in mediating the interplay between wound healing and the structural and compositional dynamics of skin microbiota remains unexplored. This is a notable gap, given that bacterial interactions on the skin surface are critically involved in the pathophysiology of skin wounds and may govern the wound healing process [16]. Polysaccharides, a class of natural macromolecular compounds, contain multiple functional groups (e.g., hydroxyl, aldehyde, and carbonyl groups) that contribute to their pronounced biological activities and structural robustness, making them indispensable in biological systems [17,18,19]. Owing to their favorable hygroscopicity, moisturizing capacity, and biological activities, they can promote wound healing and tissue growth, modulate the composition of skin microbiota, and strengthen the skin barrier defense function [20].

In parallel, the research landscape for polysaccharide wound dressings has undergone significant broadening; for example, a glucomannan/*Bletilla striata* polysaccharide composite hydrogel reported by Hao et al. promoted healing by suppressing the TNF-α/NF-κB pathway and upregulating IL-10 [21]. Despite the broad spectrum of biological activities demonstrated by polysaccharides in recent studies, the vast majority of research has centered on their basic anti-inflammatory or moisturizing functions. To date, no study has systematically placed them within a multidimensional “microbiota–immunity–repair” framework, nor has the mechanism by which polysaccharides modulate the skin microbiota to influence the dynamic wound-healing process been elucidated [22]. This study validated the wound-healing efficacy of HUPP in a full-thickness skin defect model. More importantly, it is the first to position HUPP within a multidimensional “microbiota-immunity-repair” framework, revealing that HUPP specifically remodels the wound microbiota by markedly reducing the abundance of detrimental bacteria such as *Staphylococcus*. Concurrently, HUPP regulates both local and systemic immune balance, evidenced by the downregulation of interleukin-6 (IL-6) and upregulation of interleukin-10 (IL-10) and transforming growth factor-beta (TGF-β). These findings transcend the traditional view of polysaccharides as having only broad biological activities and define HUPP as a potential “microbiota-immune” modulator.

In summary, the academic significance of this study lies in opening a new mechanistic perspective centered on “microbiota regulation” for the application of HUPP, and in providing original experimental evidence and theoretical support for the development of pro-healing materials that are distinct from conventional growth factor-based therapies.

## 2. Materials and Methods

### 2.1. Materials

*Hylocereus undatus* were purchased from Guangdong Meichen Biotechnology Co., Ltd. (Zhanjiang, China). Sulfuric acid (95–98%, GR grade) was purchased from Shanghai Lingfeng Chemical Reagent Co., Ltd. (Shanghai, China). Citric acid monohydrate (≥99.5%, AR grade), anhydrous ethanol (≥99.7%, AR grade), chloroform (≥99%, AR grade), isopropanol (≥99.5%, AR grade), sodium chloride (NaCl, ≥99.5%, AR grade), sodium hydroxide (NaOH, ≥98%, AR grade), sodium acetate (NaAc, ≥99%, AR grade), and potassium bromide (KBr, ≥99%, FT-IR grade) were purchased from Sinopharm Chemical Reagent Co., Ltd. (Shanghai, China). Trifluoroacetic acid (TFA, ≥99%, HPLC grade) and sodium azide (NaN_3_, ≥99.5%, AR grade) were purchased from Aladdin Biochemical Technology Co., Ltd. (Shanghai, China). Sodium nitrate (NaNO_3_, ≥99%, AR grade) was purchased from Macklin Biochemical Co., Ltd. (Shanghai, China). DEAE-52 cellulose (preswollen, for column chromatography) was purchased from Whatman (Maidstone, UK). Commercial hemostatic powder (Celox^®^) was purchased from Qingdao Meijin R&D Co., Ltd. (Qingdao, China). Gelatin hemostatic sponge was purchased from Guangzhou Fukang Medical Equipment Co., Ltd. (Guangzhou, China). Anti-Ki-67 antibody and anti-CD-31 antibody were purchased from Servicebio Technology Co., Ltd. (Wuhan, China). The E.Z.N.A.^®^ Soil DNA Kit was purchased from Shanghai Majorbio Biomedical Technology Co., Ltd. (Shanghai, China). Water was obtained from a Milli-Q Plus ultrapure water system (Millipore, Burlington, MA, USA). All other chemicals used in this study were of analytical reagent grade and used without further purification. Chemicals specific to analytical procedures are described in the corresponding method sections below.

### 2.2. Preparation of HUPP

Ultrasound-assisted extraction (UAE) enhances mass transfer and the extraction of target compounds via unique acoustic cavitation and mechanical effects. Its primary advantage is enabling the extraction of compounds at low temperatures. Compared with other extraction techniques, UAE reduces energy consumption and solvent usage. HUPP was extracted using a previously established ultrasonic extraction method with slight modifications [23]. Briefly, the peel of *Hylocereus undatus* was thoroughly washed, dried in a hot-air oven at 50 °C for 4 h, ground, and sieved through a 60-mesh sieve to remove impurities. Subsequently, distilled water was added to the powder at a solid-to-liquid ratio of 30:1 (*v*/*w*, mL/g), and the mixture was placed in a water bath with magnetic stirring at a controlled temperature of 90 °C for 2 h. UAE was performed using an ultrasonic device at 85 °C with a solid-to-liquid ratio of 1:30 (g/mL) for 32 min. During extraction, citric acid was added to maintain the pH at 2.5. The suspension was centrifuged at 5000× *g* for 10 min, and the resulting supernatant was concentrated using a rotary evaporator. Anhydrous ethanol was added to the concentrated solution at a ratio of 30:1 (*v*/*v*), and the mixture was incubated at 4 °C overnight (12 h) to allow complete precipitation. The precipitate was collected by centrifugation at 5000× *g* for 10 min. This process was repeated once, and the white flocculent precipitates were combined. The combined precipitate was mixed with 5 mL of Sevag reagent (chloroform: isopropanol, 4:1, *v*/*v*) for deproteinization. The upper aqueous layer (polysaccharide solution) was collected. Finally, the polysaccharide solution was purified by DEAE-cellulose chromatography. The polysaccharide solution was loaded onto a DEAE-52 cellulose column pre-equilibrated with three column volumes of distilled water. Following sample loading, the column was eluted sequentially with distilled water. Subsequently, the bound fractions were eluted with 0.5 mol/L NaCl aqueous solution and collected. The purified HUPP solution was placed in a dialysis bag and dialyzed at 4 °C for 24 h, followed by freeze-drying to obtain the final HUPP product. The freeze-dried polysaccharide was accurately weighed, and the yield was calculated using the following formula: Yield (%, *w*/*w*) = (mass of freeze-dried polysaccharide/mass of dragon fruit peel powder) × 100%. Extractions were performed independently in triplicate, and the yield is expressed as the mean ± standard deviation (SD).

### 2.3. Analysis of Monosaccharide Composition

The polysaccharide sample was hydrolyzed with 1 mL of 2 mol/L trifluoroacetic acid (TFA) at 121 °C for 2 h to ensure complete hydrolysis. Excess TFA was removed by co-evaporation with methanol three times under a stream of nitrogen. The dried sample was dissolved in distilled water, and ion chromatography was performed using a Thermo ICS 5000+ system (Thermo Fisher Scientific, Waltham, MA, USA) with high-performance anion-exchange chromatography coupled with pulsed amperometric detection (HPAEC-PAD) [24,25]. Analyze and detect monosaccharide components using multiple electrochemical detectors, including ultraviolet detectors, evaporative light-scattering detectors, differential refractive index detectors, electrochemical detectors, and conductivity detectors. A Dionex™ CarboPac™ PA20 column (150 × 3.0 mm, 10 μm) was used under the following conditions: injection volume was 5 μL. Mobile phase A (H_2_O), mobile phase B (0.1 M NaOH), mobile phase C (0.1 M NaOH, 0.2 M NaAc), flow rate 0.5 mL/min, column temperature 30 °C; Elution gradient: 0 min Phase A/Phase B/Phase C (95:5:0, V/V/V), 26 min Phase A/Phase B/Phase C (85:5:10, V/V/V), 42 min Phase A/Phase B/Phase C (85:5:10, V/V/V), 42.1 min Phase A/Phase B/Phase C (60:0:40, V/V/V), 52 min A/B/C (60:40:0, V/V/V), 52.1 min A/B/C (95:5:0, V/V/V), 60 min A/B/C (95:5:0, V/V/V). Calculate monosaccharide content based on calibration curves (peak area vs. concentration) for each monosaccharide standard. Monosaccharide contents were calculated based on the calibration curves (peak area vs. concentration) of the corresponding monosaccharide standards. All measurements were performed in triplicate.

### 2.4. X-Ray Diffraction Analysis

The freeze-dried HUPP sample was ground into a fine powder and stored in a desiccator at room temperature (25 ± 1 °C) until analysis. X-ray diffraction (XRD) patterns were recorded using an X’Pert Pro diffractometer (PANalytical B.V., Almelo, The Netherlands) with Cu-Kα radiation (λ = 0.15406 nm) operated at 40 kV and 40 mA (1600 W). The X-ray intensity was measured with a NaI scintillation counter over a 2θ range of 5–60°, a step size of 0.02°, and a scan speed of 4°/min. Each sample was independently prepared in triplicate, and each preparation was scanned three times; representative diffractograms are shown. Data were processed using HighScore Plus version 5.0, and the crystallinity index was calculated with Jade 6.0. All results are representative of three independent experiments. All results are representative of three independent experiments.

### 2.5. Molecular Weight Distribution Measurement

The molecular weight distribution of HUPP was determined using gel permeation chromatography coupled with refractive index and multi-angle laser light scattering (GPC-RI-MALS) [26]. A standard curve was plotted using dextran standards with different molecular weights (3620, 4660, 12,600, 20,000, 40,000, 50,000, 63,300 Da). The molecular weight distribution of HUPP was determined using a GPC-RI-MALS system (Wyatt Technology, Goleta, CA, USA). Accurately weighed freeze-dried HUPP powder (10.0 mg) was dissolved in 10.0 mL of the mobile phase to yield a 1.0 mg/mL solution. The solution was gently stirred in a water bath at 60 °C for 30 min and then filtered through a 0.45 μm membrane filter. Chromatographic conditions were as follows: concentration signals were recorded with a refractive index detector, and light scattering signals were collected with an 18-angle multi-angle laser light scattering (MALS) detector; the columns were Ohpak SB-805 HQ (300 × 8 mm) and Ohpak SB-803 HQ (300 × 8 mm) connected in series; Column temperature: 45 °C Injection volume: 100 μL Mobile phase A (0.02% NaN_3_, 0.1 M NaNO_3_) Flow rate: 0.6 mL/min Elution gradient: Isocratic for 75 min. Detect the concentration information of the sample based on its refractive intensity, utilize a multi-angle laser light scattering instrument to detect the light scattering information of macromolecules, and calculate the molecular weight corresponding to each component using the Mark-Houwink Equation [26]. Prior to sample loading, the sample must be dissolved in 0.1 M NaNO_3_ aqueous solution (containing 0.02% NaN_3_, *w*/*v*) and filtered through a 0.45 μm pore size filter. The sample loading volume is 20 μL. A calibration curve was constructed by plotting retention time on the abscissa against the molar mass (g/mol) of the standards on the ordinate. The molecular weight of the sample was subsequently calculated by substituting its elution time into the derived equation. All measurements were performed in triplicate.

### 2.6. Thermal Performance Analysis

The thermal decomposition temperature and moisture content of HUPP were determined using a simultaneous thermal analyzer (NETZSCH STA 449 F3 Jupiter, Hamburg, Germany). Accurately weighed freeze-dried HUPP powder (5.0 ± 0.1 mg) was placed in an alumina crucible, with an empty crucible serving as the reference. The sample was heated from 45 °C to 800 °C at a heating rate of 10 °C/min under a nitrogen atmosphere at a flow rate of 50 mL/min. Thermogravimetric analysis (TGA) and differential scanning calorimetry (DSC) curves were recorded simultaneously. The moisture content was calculated from the mass loss over the temperature range of 100–150 °C.

### 2.7. Fourier Transform Infrared Spectroscopy (FT-IR) Analysis

The chemical structure and functional groups of HUPP were characterized using a Fourier transform infrared spectrometer (Thermo Scientific™ iS10, USA) equipped with a Smart iTR diamond ATR accessory. Briefly, accurately weighed dry HUPP powder (2.0 ± 0.5 mg) and spectroscopic-grade potassium bromide (KBr, 200 ± 5 mg) were thoroughly ground and mixed in an agate mortar and then pressed into a translucent pellet. Spectra were recorded over the wavenumber range of 4000–400 cm^−1^ at a resolution of 4 cm^−1^ with 32 scans. Before each measurement, a background spectrum was acquired using a blank KBr pellet to subtract the background interference. Three independent pellets were prepared for each sample; each pellet was scanned three times, and the final spectrum represents the average of nine scans. Baseline correction and normalization were performed using OMNIC 9.0 software.

### 2.8. Morphological Analysis

The surface morphology of HUPP was examined by scanning electron microscopy (SEM). Freeze-dried HUPP powder was evenly dispersed onto carbon conductive tape, and loose particles were removed by gently blowing with a rubber air blower. To enhance electrical conductivity and minimize charging effects during SEM analysis, the sample was sputter-coated with gold using a Q150T sputter coater (Quorum, London, UK) at a current of 20 mA for 60 s, yielding a gold layer approximately 10 nm thick. SEM images were acquired using a TESCAN MIRA 3 LMU microscope operating in high-vacuum mode at an accelerating voltage of 15 kV and a working distance of 10–15 mm. Secondary electron images were recorded at magnifications of 2500×, 3200×, and 6000×. Multiple fields of view were examined and imaged for each sample.

### 2.9. Animal Experimentation

In vivo experiments were performed using C57BL/6 mice (8 weeks old), which passed the Guangdong Province Laboratory Animal Quality Qualification Certificate and were purchased from Zhuhai Biotest Biotechnology Co., Ltd. (Zhuhai, China). The mice were housed individually in ventilated cages with 5 rats per cage, maintained primarily at a controlled temperature of 23 ± 2 °C, relative humidity of 50 ± 10% in a 12 h/12 h light/dark cycle (light from 8:00 a.m. to 8:00 p.m.; dark from 8:00 p.m. to 8:00 a.m.). Food and water were provided ad libitum.

After one week of acclimatization, the mice were randomly divided into three groups using a random number table: a blank control group (CON group, *n* = 30), a HUPP treatment group (HUPP group, *n* = 30), and a positive control group (POS group, *n* = 30). A full-thickness skin wound model was then established. Under intraperitoneal anesthesia, the dorsal hair was shaved, and a circular full-thickness skin wound with a radius of 5 mm was created using surgical scissors. The wound in the CON group was treated with distilled water, whereas the HUPP group received HUPP (200 mg/kg/day). All treatments were administered by a researcher who was blinded to the subsequent assessments. The dose of HUPP used in this study (200 mg/kg/day) was selected based on the following report: Zhang et al. demonstrated that Sanguisorba officinalis polysaccharide administered intragastrically at 200 mg/kg significantly promoted burn wound healing in mice [27]. Although the aforementioned study employed oral administration, similar effective dose ranges have been observed for various natural products via both oral and topical routes—for example, Solanum xanthocarpum extract exhibited maximal wound healing efficacy both when administered orally at 200 mg/kg and when applied topically as a 10% gel [28]. Given that HUPP is a purified polysaccharide possessing a porous, sponge-like microstructure, it is theoretically expected to exhibit stronger bioactivity; hence, a topical dose of 200 mg/kg is considered reasonable. Recombinant human epidermal growth factor (rhEGF) gel (200 mg/kg/day) was selected as the positive control based on the following rationale: EGF is a well-recognized wound-healing promoter with a clearly defined mechanism of action, capable of directly stimulating epithelial cell proliferation and migration. It has been widely applied in the clinical management of burn wounds and chronic ulcers and is routinely used as a standard reference to evaluate the pro-healing capacity of novel dressings or agents in basic research [29,30,31,32]. Comparing HUPP with this clinically validated positive control allows for a more rigorous assessment of its relative therapeutic potential in wound repair.

Mice were euthanized on days 3, 6, 9, and 12 post-treatment. The number of experimental mice in each group at each time point is shown in Table 1. Blood was collected from the orbital sinus, centrifuged at 600× *g* for 15 min at 4 °C to obtain serum samples, which were stored at −20 °C. Skin tissue was harvested and immediately frozen at −80 °C for subsequent analysis. All animal treatments and experiments were conducted strictly in accordance with the National Research Council’s Guide for the Care and Use of Laboratory Animals. All experimental protocols complied with the ARRIVE guidelines. Animal experiments and procedures were approved by the Animal Care and Welfare Committee of Guangdong Ocean University (Approval No.: GDOU-LAE-2024-028).

### 2.10. Determination of Body Weight Change Rate and Organ Coefficients

After modeling, the body weights of the mice were recorded on the modeling day and days 3, 6, 9, and 12. The kidneys, spleens, and livers were collected and precisely weighed using an analytical balance. Organ coefficients were calculated as 100 × organ weight/body weight (100 × g/g).

### 2.11. Measurement of Wound Closure Rate

On days 0, 3, 6, 9, and 12, the wounds of each group of mice were photographed using a ruler and black circular hole cover cloth to mark fixed points for determining the daily reduction in wound area. After each photograph is taken for documentation, the HUPP group was treated with fresh HUPP, the CON group must be coated with saline solution, and the POS group must be coated with recombinant human epidermal growth factor gel. Finally, gauze and adhesive tape must be applied.

The wound closure rate calculation formula is as follows:

(1) $$\mathrm{Wound}\;\mathrm{closure}\;\mathrm{rate}\;(\%)\;=\;\left(1\;-\;\frac{A_{t}}{A_{0}}\right)\;\times \;100$$

A_0_: area of wound on the day of modeling;

A_t_: Remaining area of wound on day t.

### 2.12. Histological Assessment

Skin tissue from the wound site was fixed with 4% paraformaldehyde, embedded in paraffin, and examined for histopathological changes. Paraffin-embedded skin tissue was manually sectioned into 4.5 μm thick slices using a microtome. After dewaxing, the sections were stained with hematoxylin and eosin (H&E) and Masson’s trichrome staining. Wound healing progression and granulation tissue growth were observed under a microscope.

### 2.13. Microbial 16s rRNA Sequencing

Genomic DNA was extracted from mouse skin wound tissue samples using the FastPure Soil DNA Isolation Kit (Magnetic bead, MJYH, Shanghai, China). The V3-V4 region of the bacterial 16S rRNA gene was amplified using a GeneAmp 9700 Thermal Cycling PCR System (ABI, Foster City, CA, USA) with primers 338F (ACTCCTACGGGGAGGCAGCAG) and 806R (GGACTACHVGGGTWTCTAAT). The amplified DNA samples were sequenced by Shanghai Majorbio Biomedical Technology Co., Ltd. using the Illumina MiSeq PE300 platform.

Sequences with 97% similarity were clustered into operational taxonomic units (OTUs) using UPARSE (version 7.1). All OTUs were taxonomically classified against the SILVA 138 reference 16S rRNA database with the Ribosomal Database Program (RDP) classifier at a confidence threshold of 70%. The relative abundances of microbial communities at various taxonomic levels were determined based on the taxonomic distribution of OTUs. After quality filtering, the number of sequencing reads per sample ranged from 30,000 to 80,000. To ensure comparable sequencing depth across samples, all samples were rarefied to 50,000 reads per sample. Intergroup differences in β-diversity were evaluated by permutational multivariate analysis of variance (PERMANOVA) using the Bray–Curtis dissimilarity matrix with 9999 permutations. Alpha and β-diversity analyses were performed with QIIME 2 software package (version 2024.10) based on the OTU data. Differentially abundant microbial taxa among treatment groups were identified using linear discriminant analysis effect size (LEfSe). The microbial community composition and differential abundance results were visualized with Circos plots and LEfSe bar plots/cladograms.

### 2.14. Immunofluorescence Analysis

To further assess wound healing, inflammation, and angiogenesis, immunofluorescence staining for Ki-67 (1:500, GB111141, Servicebio) and CD-31 (1:200, GB15063, Servicebio) was performed simultaneously on skin wound tissues collected on days 6 and 12. For immunofluorescence staining, sections were sequentially deparaffinized in environmentally friendly dewaxing solution I (10 min), dewaxing solution II (10 min), dewaxing solution III (10 min), followed by rehydration in graded ethanol: absolute ethanol I (5 min), absolute ethanol II (5 min), absolute ethanol III (5 min), and a final rinse in distilled water. Antigen retrieval was then performed, after which the sections were allowed to cool naturally. Slides were washed three times (5 min each) with PBS (pH 7.4) on a rocking platform. After the sections were slightly shaken dry, a hydrophobic barrier was drawn around the tissue using a PAP pen, and the sections were blocked with BSA for 30 min. They were then incubated overnight at 4 °C with primary antibodies against Ki-67 and CD-31. Following three washes with PBS (5 min each), the corresponding secondary antibodies (Alexa Fluor 488-conjugated goat anti-rabbit IgG and Alexa Fluor 488-conjugated goat anti-mouse IgG) were applied and incubated in the dark at room temperature for 50 min. The sections were counterstained with DAPI, and after quenching tissue autofluorescence, they were coverslipped. Fluorescence images were captured using an upright fluorescence microscope (Nikon Eclipse E100, Nikon Corporation, Tokyo, Japan).

All quantitative analyses were conducted in a blinded manner. Specifically, a researcher not involved in the experimental grouping randomly coded all images and concealed the group assignments until the analysis was complete. For each wound section, five non-overlapping fields were imaged using identical acquisition parameters. The fluorescence intensities of Ki-67 and CD-31 were quantified using ImageJ (version 1.53q) software software. After subtracting the background fluorescence measured in an unstained area, the mean fluorescence intensity (MFI) of each field was determined. The average MFI of the five fields was calculated as the representative value for each sample. Two independent observers blinded to group allocation performed the quantitative analyses, and their averaged results were used for statistical analysis.

### 2.15. Measurement of Inflammatory Cytokine Levels

Blood samples were collected from the orbital sinuses of mice on days 3, 6, 9, and 12. The samples were allowed to clot at 25 °C for 10 min, then centrifuged at 6000× *g* for 15 min at 4 °C to obtain serum. Serum levels of cytokines, including IL-6, IL-10, and TGF-β, were measured using specific enzyme-linked immunosorbent assay (ELISA) kits according to the manufacturer’s instructions.

### 2.16. Statistical Analysis

All experimental data obtained are expressed as the mean ± standard deviation (SD) from at least three independent replicates. Results between the control group and experimental groups were compared using analysis of variance (ANOVA) and Bonferroni multiple comparisons. Statistical analysis was performed using GraphPad Prism (version 10.1.2) and Microsoft Excel 2021. Perform image analysis using ImageJ version 1.53t. Statistical significance was considered at * *p* < 0.05, ** *p* < 0.01, *** *p* < 0.001, and **** *p* < 0.0001.

## 3. Results and Discussion

### 3.1. Chemical Properties of HUPP

**Figure 1:**
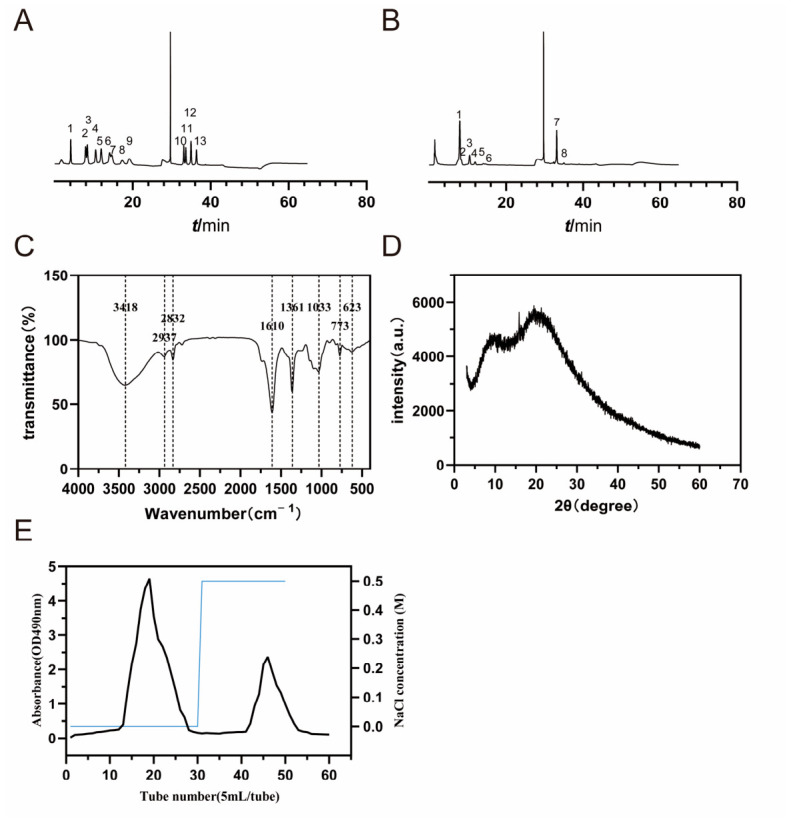
The chemical properties of HUPP. (**A**) The ion chromatogram of the standard sample, and the response peaks of each monosaccharide are marked with numbers in the figure. (**B**) The ion chromatogram of the sample, with the response peaks of each monosaccharide marked by numbers in the figure. (**C**) The Fourier infrared spectrogram of HUPP, with the corresponding wavelengths of each characteristic peak marked on the graph. (**D**) XRD spectra. (**E**) DEAE-cellulose elution profile of HUPP.

HUPP was obtained from the peel of *Hylocereus undatus* after ultrasound-assisted aqueous extraction followed by ethanol precipitation, deproteinization using the Sevag method, and purification by DEAE-cellulose column chromatography. The yield of purified HUPP was 12.66%. To ensure reproducibility, detailed protocols covering all critical parameters for extraction, deproteinization, column chromatography, and freeze-drying are provided in Section 2.2 (Preparation of HUPP). The monosaccharide composition of HUPP was determined by ion chromatography [33]. The ion chromatograms of the monosaccharide standards and the HUPP sample are presented in Figure 1A,B. HUPP was predominantly composed of galacturonic acid (Gal-UA), rhamnose (Rha), galactose (Gal), arabinose (Ara), glucose (Glc), mannose (Man), glucuronic acid (Glc-UA), and xylose (Xyl), with a Gal-UA to Rha ratio of 0.78:1. The monosaccharide contents were as follows: Gal-UA, 243.4 μg/mg; Rha, 262.6 μg/mg; Gal, 11.0 μg/mg; Ara, 23.05 μg/mg; Glc, 18.8 μg/mg; while Man (8.8 μg/mg), Glc-UA (8.3 μg/mg), and Xyl (7.0 μg/mg) were present in minor amounts. The specific information on the composition of monosaccharides is shown in Table 2. The monosaccharide composition directly influences the biological activities and physicochemical properties of polysaccharides, and these results suggest that HUPP is an acidic heteropolysaccharide [34,35]. Gal-UA and Rha, which commonly co-exist in polysaccharides, are capable of synergistically potentiating immunomodulatory effects [36,37]. It is speculated that HUPP may possess biological activities such as immunomodulation, antioxidant properties, and anti-inflammatory effects [38,39].

FT-IR spectroscopy was used for structural analysis of HUPP as shown in Figure 1C [40,41]. The broad peak in the 4000–1300 cm^−1^ range is used to analyze characteristic functional groups, while the region below 1000 cm^−1^ provides information on the structural conformation of the polysaccharides [42]. The broad and intense absorption band between 3400 and 3200 cm^−1^ is attributed to O–H stretching vibrations arising from intra- and intermolecular hydrogen bonding, a hallmark feature of carbohydrate compounds. This directly accounts for the favorable water solubility and hygroscopicity of HUPP, providing a structural basis for its application in a moist wound-healing environment. However, the broad profile of this peak also indicates the presence of abundant free hydroxyl groups, although it does not allow differentiation between the hydration states of crystalline and amorphous regions. This observation is consistent with the amorphous, low-crystallinity structure of HUPP revealed by XRD, since amorphous polysaccharides typically expose more hydroxyl groups, thereby enhancing hydrophilicity [43]. The weak absorption band at 2930–2880 cm^−1^ corresponds to the C–H stretching vibrations of methylene (–CH_2_–) and methine (–CH–) groups on the sugar ring. The low intensity of this peak indicates that the hydrophobic moieties of the sugar backbone are relatively minor, further supporting the overall hydrophilic nature of HUPP. The absorption peak in the region of 1600–1650 cm^−1^ may originate from the asymmetric stretching vibration of carboxylate groups (COO^−^) or the O–H bending vibration of adsorbed water, suggesting the possible presence of uronic acids. Based on the monosaccharide composition analysis, which confirmed a high galacturonic acid content, this peak is assigned to the overlapping contributions of the asymmetric stretching vibration of COO^−^ and the O–H bending of adsorbed water. The weak absorption band around 1400 cm^−1^ is attributed to the symmetric stretching vibration of carboxylate groups (COO^−^). Together with the peak at 1600–1650 cm^−1^, this pairing further corroborates the presence of uronic acids. The strong and broad absorption band in the range of 1100–1000 cm^−1^ corresponds to the fingerprint region of pyranose rings, predominantly arising from the stretching vibrations of glycosidic bonds (C–O–C) and hydroxyl groups (C–O–H). The broad nature of this band indicates complex coupling and overlapping vibrations of the sugar rings. Compared with typical β-configured polysaccharides reported in the literature, HUPP did not display sharp, well-resolved peaks in this region, implying that the glycosidic linkages are predominantly β-type but also contain a minor proportion of α-linkages. [44]. Weak absorption bands were observed in the fingerprint region below 1000 cm^−1^, which generally reflect conformational information of the sugar chains. A characteristic absorption peak corresponding to β-type glycosidic linkages was detected at approximately 890 cm^−1^, indicating that the sugar residues in HUPP are predominantly linked via β-configuration. In contrast, α-configured glycosidic bonds typically show an absorption band near 840 cm^−1^, which was not clearly observed in the HUPP spectrum. Therefore, the glycosidic linkages in HUPP are overwhelmingly of the β-configuration.

Crystallinity was verified using an X’ Pert Pro X-ray diffractometer (PANalytical B.V., Almelo, The Netherlands). As shown in Figure 1D, a broad diffraction peak appears at an angle of 8°, typically corresponding to larger spacings between polysaccharide chains. This indicates a loose structure without regular crystalline arrangement [25]. A broad peak appears at 20°, indicating that the material exhibits only short-range order and long-range disorder internally. Within the small range of a few sugar units, there may be some degree of regularity. However, on a larger scale, the molecular chains are disordered and randomly arranged, unable to form a lattice structure capable of producing sharp diffraction peaks [45,46,47]. To validate this conclusion, the polysaccharide crystallinity was calculated as 8.94% based on the total area and non-crystalline area. This indicates that the crystallinity of HUPP is relatively low, suggesting they primarily exist in an amorphous state with an amorphous structure, consistent with the FT-IR analysis results [48]. Amorphous structures typically indicate that the polysaccharide possesses good solubility, water-holding capacity, and potentially enhanced biological activity, which is a positive sign for its functional applications.

### 3.2. Physical Properties of HUPP

Molecular weight is a critical determinant of polysaccharide bioactivity. Using ASTRA 6.1 software to process chromatographic data, the weight-average molecular weight (Mw), number-average molecular weight (Mn), peak molecular weight (Mp), polydispersity index (PDI), and chain conformation—all important physical characteristics of the polysaccharide—were determined [25]. The specific molecular weight information is shown in Table 3. The chromatogram of HUPP displayed a characteristically asymmetric peak with pronounced tailing [15,46,49]. The Mw of HUPP was 227 ± 6.36 kDa, the Mn was 113.2 ± 3.08 kDa, and the PDI was 2.01 ± 0.08, indicating that HUPP is a heterogeneous polysaccharide, consistent with the characteristic features of natural plant polysaccharides [42,49]. The markedly lower Mn relative to Mw indicates that a considerable number of low-molecular-weight fractions are present in the sample, which may arise from degradation during extraction or from inherent differences in the degree of polymerization. During the extraction of plant polysaccharides, varying degrees of degradation can occur, and their biosynthesis naturally produces molecules with different degrees of polymerization [46]. The radius of gyration (Rg) describes the dispersion of mass around the center of mass of a polymer chain. Intriguingly, the high-molecular-weight fractions of HUPP exhibited a smaller Rg than some low-molecular-weight fractions. This anomalous trend suggests that the high-molecular-weight fraction adopts a highly branched, compact spherical structure, whereas the low-molecular-weight fraction exists as linear or lightly branched random coils. Because low-molecular-weight components may be linear or only slightly branched, they occupy a larger space per unit molecular mass, consistent with an extended linear or random-coil conformation rather than a compact globule. The anomalous relationship, with the weight-average radius of gyration (Rw) being smaller than the number-average radius (Rn), together with the small absolute dimensions, further confirms that the high-molecular-weight portion of the polysaccharide possesses a highly branched, compact architecture. Its conformation more closely approximates a branched sphere than a random coil or a rigid rod.

**Figure 2:**
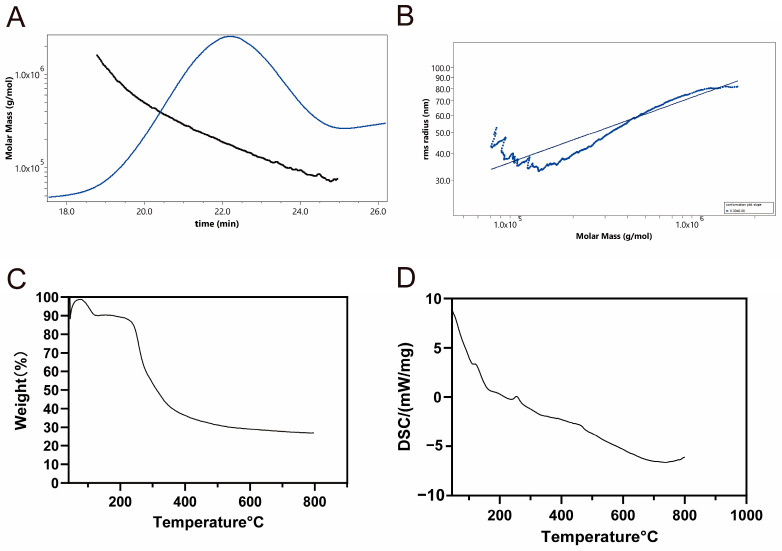
The physical properties of HUPP. (**A**) The gel chromatogram of HUPP; the black curve represents the absolute molar mass (Molar Mass 1), and the blue curve represents the signal intensity (*n* = 3). (**B**) The graph shows the relationship between the root mean square rotational radius of HUPP and the molecular weight. The blue curve represents the measured RMS radius values of different molecular weight components (*n* = 3). (**C**) DSC thermogram of HUPP. (**D**) TGA thermogram of HUPP.

The thermal properties of a material are also critical determinants of its application. Therefore, HUPP was further characterized by TGA and DSC. The weight loss profile of the polysaccharide, as shown by the TGA curve in Figure 2C, can be divided into three stages. The first stage (45–100 °C) corresponds to the loss of adsorbed water and its evaporation. The second stage (100–400 °C) represents the main thermal decomposition phase, during which the molecular chains begin to break down and decompose into small molecules. In the third stage (400–800 °C), the mass decreases gradually and eventually stabilizes; the residual material, typically ash, exhibits high thermal stability.

To further explore the thermal transitions of HUPP, the DSC curve was analyzed. The thermal process can be divided into three stages. In the initial stage (45–250 °C), the DSC trace remained relatively flat while the mass percentage decreased rapidly from 100% to 87%, reflecting the evaporation of adsorbed and bound water. This is consistent with the strong, broad O–H stretching band at 3400 cm^−1^ in the FT-IR spectrum, confirming the highly hydrophilic nature of HUPP. The main exothermic stage (250–500 °C) was characterized by a sharp decline in the DSC curve and a broad exothermic peak, corresponding to backbone cleavage, depolymerization, and subsequent oxidative combustion. During this stage, the mass percentage continued to decline from 87% to 32%. In the final stage (>500 °C), the exothermic reaction subsided and the heat flow returned to baseline, leaving a final char residue of 26%. No distinct glass transition was observed over the entire temperature range, which is in excellent agreement with the amorphous structure of HUPP indicated by XRD and SEM. Collectively, the thermal analysis demonstrates that HUPP possesses good thermal stability and a moisture content of 13%.

**Figure 3:**
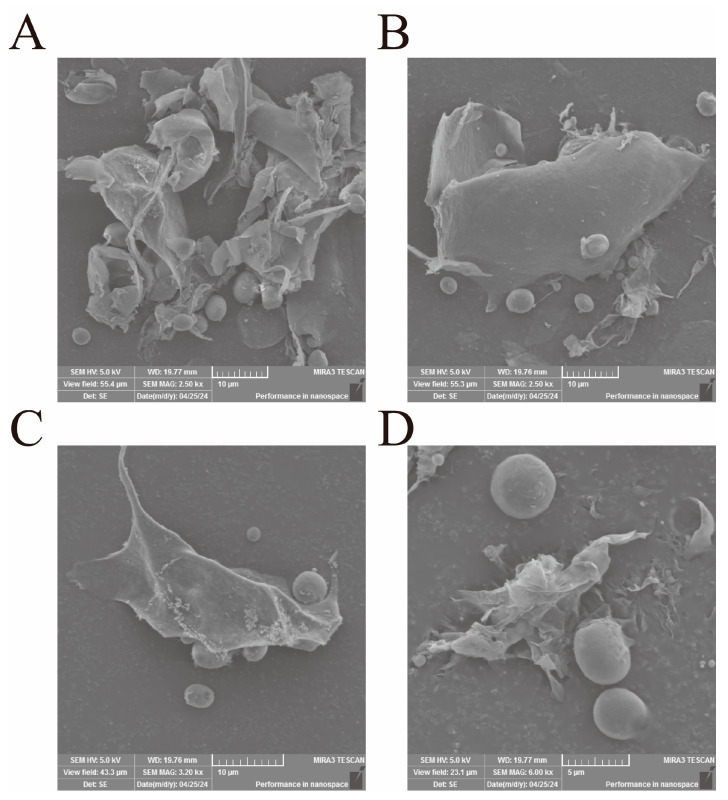
The scanning electron microscope images of HUPP. (**A**) The microscopic morphology of HUPP observed under the 2.5 k× magnification. (**B**) Another perspective of the microscopic morphology of HUPP observed under the 2.5 k× magnification. (**C**) The microscopic morphology of HUPP observed under the 3.2 k× magnification. (**D**) The microscopic morphology of HUPP observed under the 6.0 k× magnification.

To obtain structural evidence at the microscale, HUPP was examined by SEM to directly visualize its surface morphology and architecture. As shown in Figure 3, at 2500× magnification, HUPP displayed a large, continuous, amorphous film-like morphology with slight wrinkles and undulations. This smooth, continuous film-like aggregate is typically attributed to the random entanglement of highly branched polysaccharide chains in the solid state, which lack long-range order and thus form an amorphous structure. This observation, together with the absence of sharp diffraction peaks in XRD and the lack of crystalline features in FT-IR, collectively supports the low-crystallinity nature of HUPP. At 3200× magnification, a finer porous structure became evident. Pores of varying sizes were distributed within and on the surface of the film, revealing an incipient sponge-like architecture. The most plausible formation mechanism is related to the freeze-drying process: water in the sample forms ice crystals upon freezing, and after sublimation, voids are left behind; the highly branched polysaccharide chains serve as a framework that aggregates in the interstitial spaces of the ice crystals, ultimately forming the pore walls. It should be noted, however, that the size, distribution, and interconnectivity of the pores are markedly influenced by parameters such as freezing rate, sample concentration, and drying conditions. The conditions employed in this study (see Section 2.8) are not the only standard, and porosity may vary across batches. At higher magnification (6000×), detailed images clearly showed that the walls constituting the sponge-like structure were very thin and smooth, again confirming the amorphous character of the material. No crystalline or regular geometric shapes were observed, and the amorphous nature and smooth surface are consistent with the surface characteristics of branched molecules and hydrophilic groups. Taken together, the structural characterization clearly demonstrates that HUPP is an amorphous material with a porous, sponge-like microstructure. Theoretically, this porous, sponge-like microstructure of HUPP has several potential biological implications—it increases the specific surface area, facilitates the absorption of wound exudate, and provides a physical scaffold for the migration of fibroblasts and vascular endothelial cells, thereby promoting cell growth and migration, which are beneficial for wound healing.

### 3.3. Therapeutic Effects of HUPP on Skin Wounds

**Figure 4:**
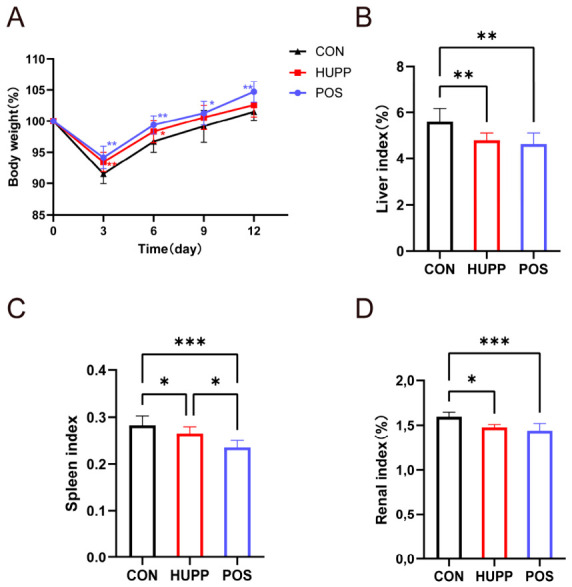
Changes in body weight and organ coefficients of mice. (**A**) Body weight. (**B**) Liver coefficient. (**C**) Spleen coefficient. (**D**) Renal coefficient of mice in each treatment group (*n* = 6). Statistical significance was considered at * *p* < 0.05, ** *p* < 0.01, *** *p* < 0.001, and **** *p* < 0.0001.

During the experimental period, no mortality, abnormal behavior, or obvious signs of toxicity were observed in any of the groups. Body weight reflects metabolic and recovery status. As shown in Figure 4A, the body weight change exhibited a trend of the POS group < the HUPP group < the CON group. On day 3 post-wounding, the rate of body weight change declined in all groups, which may be attributable to reduced food intake caused by wound-induced pain. On day 6, the rate of body weight change in the HUPP group and the POS group was significantly higher than that in the CON group, suggesting that both HUPP and the positive control may alleviate wound-induced metabolic stress without adversely affecting overall metabolism. On days 9 and 12, a significant difference was still observed between the POS group and the CON group, whereas no significant difference was found between the HUPP group and the CON group, indicating that, in the short term, HUPP can mitigate the impact of metabolic stress on body weight and exert a protective effect in mice.

**Figure 5:**
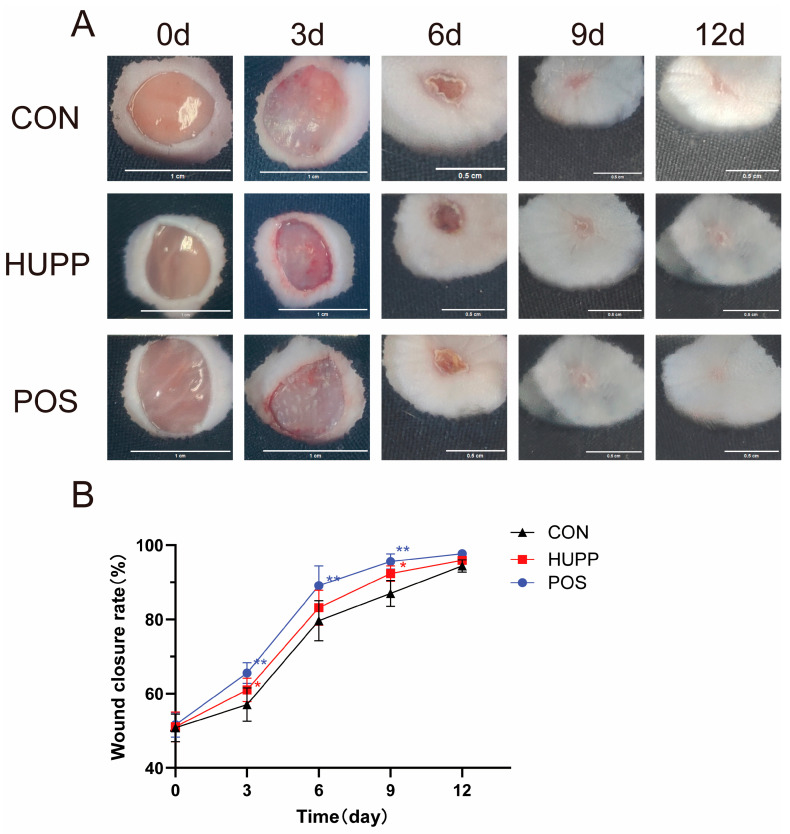
The analysis of wound healing. (**A**) Representative pictures of wounds in each group; representative photographs of wounds from CON, HUPP, and POS groups on days 0, 3, 6, 9, and 12 post-wounding. (**B**) Time course of wound closure rate, detailed animal procedures are described in Materials and Methods (*n* = 6). Statistical significance was considered at * *p* < 0.05, ** *p* < 0.01, *** *p* < 0.001, and **** *p* < 0.0001.

Meanwhile, wound areas were measured and recorded on days 3, 6, 9, and 12 of the wound-healing process, and the wound healing rates were calculated. The specific representative pictures of the wounds and the curves showing the wound healing rates are shown in Figure 5. The experimental results showed that on day 3 post-wounding, both the POS group (* *p* < 0.05) and the HUPP group (** *p* < 0.01) exhibited significant differences compared with the CON group. On day 6, the wound healing rates of the POS group and the HUPP group were both higher than that of the CON group, with the POS group reaching statistical significance (* *p* < 0.05); the wound closure speed of the CON group was markedly slower than that of the treated groups. On day 9, the wound healing rate in the HUPP group approached that in the POS group, and both the POS group (* *p* < 0.05) and the HUPP group (** *p* < 0.01) remained significantly different from the CON group. By day 12, wounds in all groups were almost completely healed. Examination of the major organs for organ index determination revealed no obvious necrosis, inflammation, or structural abnormalities.

### 3.4. HUPP Promote Wound Tissue Growth

**Figure 6:**
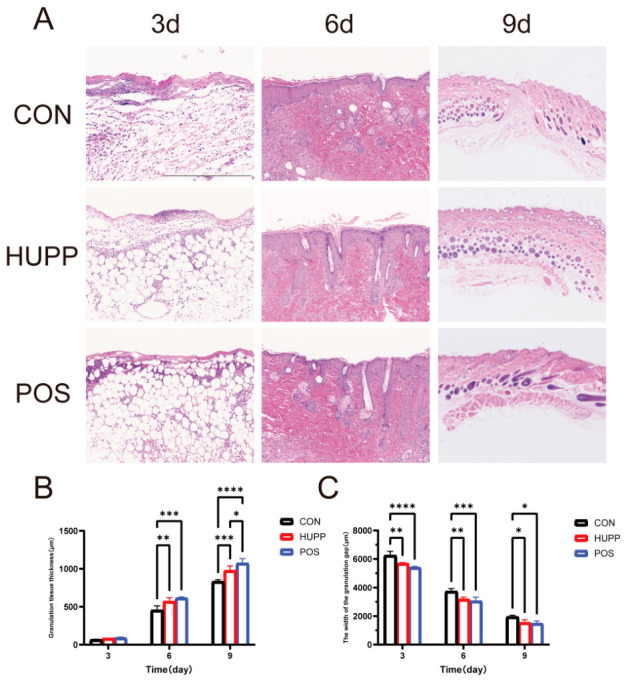
Histological analysis of skin wound tissue. (**A**) Representative histological staining images of granulation tissue in different treatment groups (CON, HUPP, POS) on the 3rd, 6th, and 9th days after modeling. (**B**) The image of granulation tissue thickness; It shows the width of the granulation tissue on the 3rd day, the 6th day and the 9th day after the model was established. (**C**) The width of the Granulation gap; It shows the length of the wound gap on the 3rd, 6th and 9th days after the model was established (*n* = 6). Statistical significance was considered at * *p* < 0.05, ** *p* < 0.01, *** *p* < 0.001, and **** *p* < 0.0001.

To further investigate the wound conditions during the inflammatory, proliferative, and remodeling phases of healing, skin sections were stained with H&E to evaluate the histological changes in wound tissue and granulation tissue formation. As shown in Figure 6A, on day 3 all groups exhibited inflammatory infiltration and edema, with distinct purplish-red inflammatory exudate and white adipose tissue beneath the epithelial layer. The granulation gap width in the CON group was significantly different from that in the HUPP group (** *p* < 0.01) and the POS group (**** *p* < 0.0001), indicating that HUPP markedly reduced the granulation gap width. On day 6, capillary lumina and bright red granulation tissue were observed in the POS group, which showed the most pronounced differentiation, with thickened granulation tissue, increased mitotic figures, and re-epithelialization characterized by epidermal cell migration from the wound edges. At this time point, the granulation gap width and granulation tissue thickness in the CON group differed significantly from those in the HUPP group (** *p* < 0.01) and the POS group (**** *p* < 0.0001), demonstrating that HUPP significantly promoted granulation tissue formation and thereby accelerated wound healing. On day 9, elongated fibrocytes were clearly visible, capillaries decreased in number, and the neo-epidermis was thickened, although undifferentiated granulation tissue was still present in the CON group. The granulation gap width in the CON group was significantly different from that in the HUPP group (** *p* < 0.01) and the POS group (** *p* < 0.01), which was consistent with the observation of incomplete granulation tissue differentiation in the CON group. Moreover, the granulation tissue thickness in the CON group differed significantly from that in the HUPP group (** *p* < 0.01) and the POS group (**** *p* < 0.0001), and a significant difference was also found between the HUPP group and the POS group (* *p* < 0.05). These results confirm that HUPP can effectively promote the reconstruction of damaged blood vessels and fibroblast differentiation in granulation tissue at the tissue level, highlighting its potential as a wound dressing [50].

**Figure 7:**
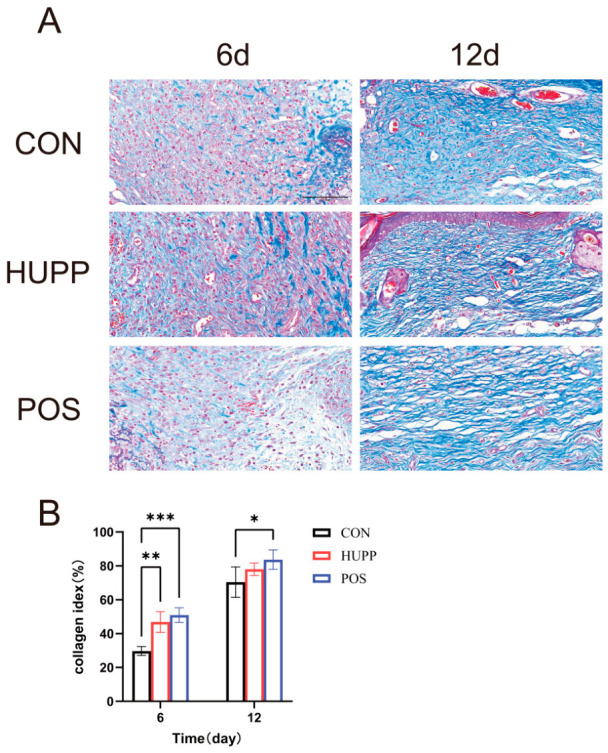
Analysis of collagen deposition proteins. (**A**) Collagen staining map. (**B**) Quantified map of collagen deposition (*n* = 6). Statistical significance was considered at * *p* < 0.05, ** *p* < 0.01, *** *p* < 0.001, and **** *p* < 0.0001.

During wound regeneration, the differentiation of fibroblasts within granulation tissue is of paramount importance [51]. The proliferation of epidermal and dermal cells marks the onset of tissue repair during wound healing. For fibroblasts, collagen deposition is their most essential function [52]. To further investigate fibroblast differentiation during wound healing, Masoon’s staining was performed to assess the extent of collagen deposition. On day 6 post-wounding, small amounts of collagen were present in all groups, but the distribution was uneven. On day 9, compared with the CON group, the HUPP group displayed more intense blue staining, with collagen distributed more uniformly and in a more organized pattern; the blue-stained area was also enlarged relative to day 6, indicating enhanced collagen synthesis and closely approaching the appearance of the POS group. As shown by the quantitative analysis of collagen deposition in Figure 7B, the collagen content in the HUPP group was similar to that in the POS group, demonstrating that HUPP can accelerate wound healing by promoting collagen synthesis.

### 3.5. Alterations in Skin Microbiota Diversity Induced by HUPP

**Figure 8:**
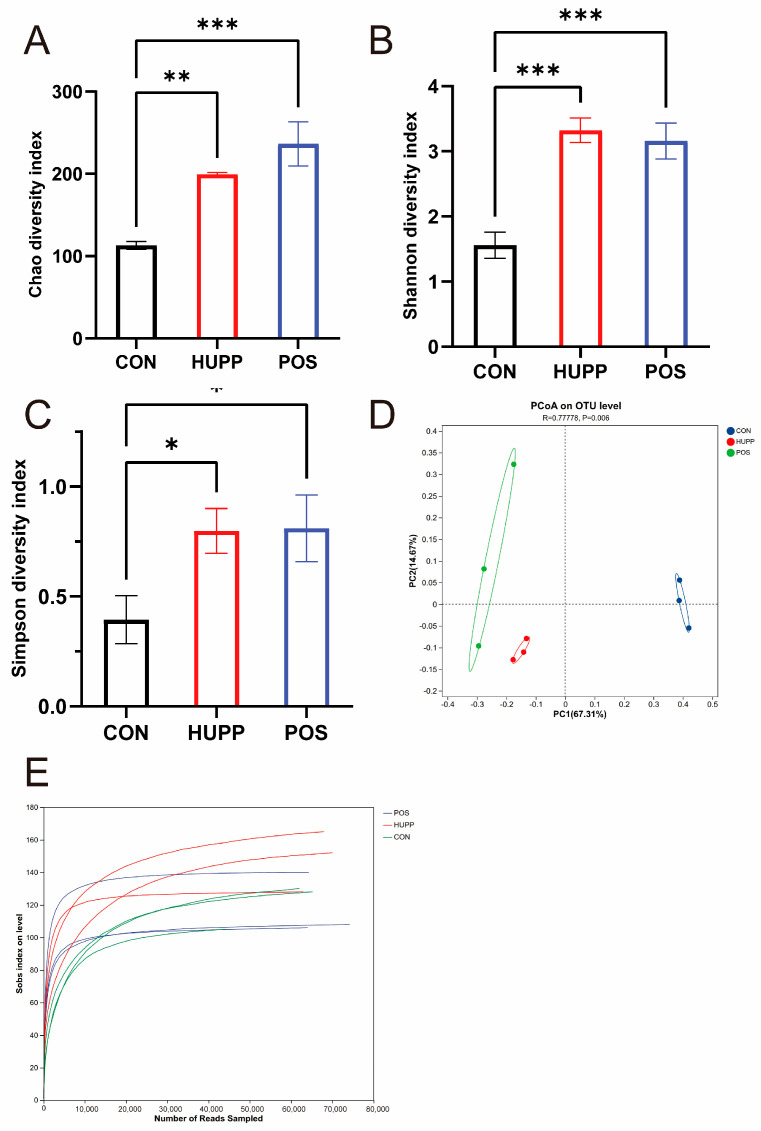
Changes in skin microbiota diversity induced by HUPP. (**A**) Chao index. (**B**) Shannon index. (**C**) Simpson index. (**D**) Skin flora PCoA assay results. Samples from different groups are indicated by different colors. (**E**) Rarefaction curves of the bacterial community (*n* = 4). Statistical significance was considered at * *p* < 0.05, ** *p* < 0.01, *** *p* < 0.001, and **** *p* < 0.0001.

In practical research, particularly in microbiome studies, α-diversity and β-diversity are the most commonly used core metrics for measuring community changes [53]. The diversity reflects species richness and evenness within a community: the Chao index measures species richness, and the Shannon index accounts for both richness and evenness. Higher values indicate greater diversity [54]. The Simpson index places greater emphasis on the dominance of dominant species; treatment with HUPP and the positive control medicine significantly increased the number of microbial species in the wound tissue (Figure 8A–C). The Shannon index for the HUPP group was significantly higher than that of the CON group, indicating that HUPP not only increases species richness but also promotes a more balanced species distribution, resulting in healthier overall diversity. The Simpson index places greater emphasis on measuring community dominance. The Simpson index showed significant differences between the HUPP, POS, and CON groups (* *p* < 0.05), with the highest value in the HUPP group indicating the lowest community dominance. These results demonstrate that HUPP helps restore microbial diversity on the skin wound surface, with effects comparable to the positive control medicine, reflecting its potential clinical relevance in treating inflammatory skin wounds.

β-diversity reflects the “between-sample” variation in microbial community structure, complementing α-diversity. Principal coordinate analysis (PCoA), in particular, captures the diversity differences among samples. PCoA based on the Bray–Curtis distance (R^2^ = 0.57, *p* < 0.05) revealed a clear separation trend among the microbial community structures of the three groups (the CON, HUPP, and POS groups). This analysis calculates the “ecological distance” between every pair of samples, which numerically quantifies the degree of similarity in their microbial composition [54,55]. A Permanova analysis was also conducted to compare the differences in the composition of the wound microbial communities among the different treatment groups. The specific information is shown in Table 4. PC1 exhibits a significant difference of 67.31%, making it the most important loading value for distinguishing the CON group from other groups. PC2 represents a significant difference of 14.67%, clearly distinguishing the HUPP group from the POS group. These results confirm that the overall microbial composition of the CON group differs significantly from that of the treatment groups, and HUPP treatment substantially reshapes the microbial community structure.

### 3.6. HUPP Modulate Skin Microbiota Composition in Wound Models

**Figure 9:**
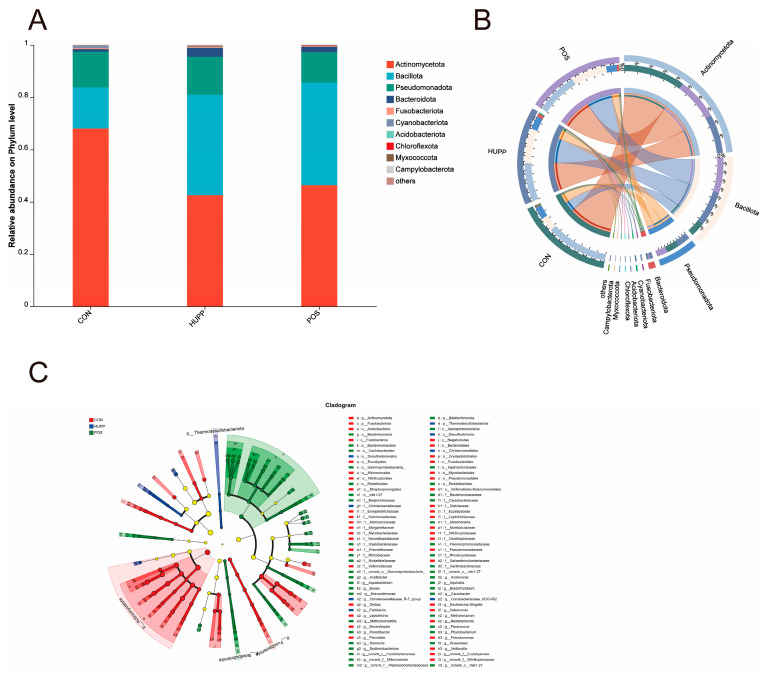
Modulation of skin microbiota composition by HUPP in a mouse wound model. (**A**) Relative abundance of mouse skin bacterial flora at the phylum level. (**B**) Distribution of microorganisms at the phylum level using Circos plots, with different groups of samples indicated by different colors (*n* = 4). (**C**) LEfSe analysis of the skin microbiota. Differentially abundant bacterial taxa among the CON, HUPP, and POS groups are shown.

**Figure 10:**
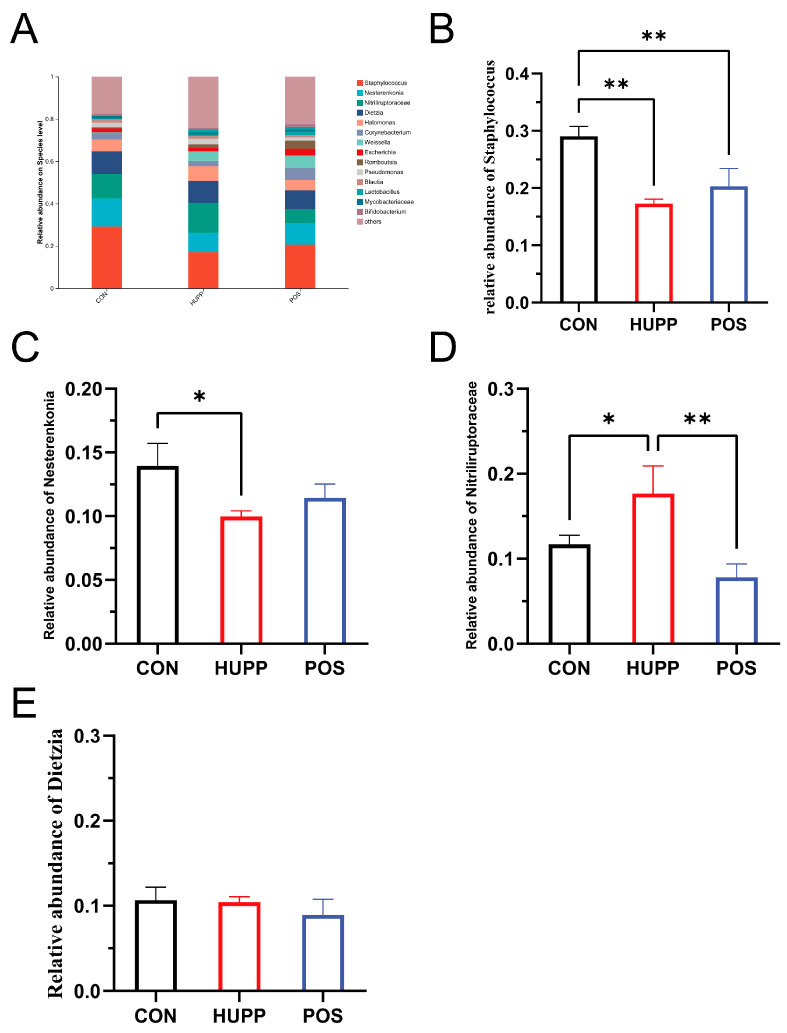
Modulation of skin microbiota composition by HUPP in a mouse wound model. (**A**) Relative abundance of mouse skin bacterial flora at genus level. (**B**) Relative abundance of *Staphylococcus*. (**C**) Relative abundance of *Nesterenkonia*. (**D**) Relative abundance of *Nitriliruptoraceae*. (**E**) Relative abundance of *Dietzia*. Different groups of samples are indicated by different colors (*n* = 4). Statistical significance was considered at * *p* < 0.05, ** *p* < 0.01, *** *p* < 0.001, and **** *p* < 0.0001.

16S rRNA gene sequencing was performed to investigate the effects of HUPP on the wound microbiota composition (Figure 9 and Figure 10). The results indicate that HUPP treatment significantly altered the community structure and composition of the wound microbiota. At the phylum level, the dominant microbial communities primarily consist of *Actinomycetota*, *Bacillota*, and *Pseudomonadota*, followed by *Bacteroidota* and others. Among these, the relative abundance of *Actinobacteria* in the CON group (68.04%) was higher than that in the HUPP group and the POS group, while HUPP treatment caused the proportion of Firmicutes to approach that of the POS group. Circos diagrams (Figure 8B) visualized the species composition relationships among the three groups: *Actinomycetota* and *Bacillota* are two common bacterial phyla found on the skin surface. *Actinomycetota* plays a crucial role on the skin by fermenting triglycerides in sebum to produce short-chain fatty acids, thereby helping maintain the skin’s surface as a weakly acidic environment. Additionally, *Bacillota* can secrete antimicrobial peptides to inhibit potential pathogens.

At the genus level (Figure 10A), HUPP treatment significantly reduced the relative abundance of *Staphylococcus* (Figure 10B). *Staphylococcus aureus* and other *Staphylococcus* spp. secrete α-toxins that directly damage epithelial cells and fibroblasts, hindering wound repair [56]; thus, the reduction in *Staphylococcus* indicates that HUPP eliminates staphylococci-induced persistent inflammation, toxin damage, and biofilm formation. *Nesterenkonia*, an opportunistic commensal that can become pathogenic under conditions such as compromised immunity or foreign body implantation, also showed reduced relative abundance in the HUPP group (Figure 10C), helping to modulate the microbial community toward a healthier state similar to the skin’s resident flora. This helps modulate the microbial community toward a healthier state more similar to the skin’s resident flora. For *Nitriliruptoraceae* (Figure 10D), a low-abundance genus, HUPP treatment may have suppressed other dominant bacterial communities, freeing up ecological niches and resources for its growth. *Dietzia* (Figure 10E) may regulate local inflammatory responses through its cell wall components or metabolites, but no significant differences were observed between treatment groups, possibly due to bidirectional regulation dependent on its abundance and specific strain. To further quantitatively identify bacterial taxa with significantly differential abundances among the treatment groups, LEfSe analysis was performed. The results are presented in the figure.

These results indicate that HUPP exerts a significant regulatory effect on the structural composition of the wound microbial community, which may be a key mechanism underlying its therapeutic efficacy. Specifically, HUPP promotes the development of the microbial community toward a structure similar to the POS group, particularly by reducing the abundance of virulent genera such as *Staphylococcus*, suggesting that HUPP exerts positive effects on the wound microenvironment through an ecological mechanism that suppresses harmful microbes and promotes beneficial ones.

### 3.7. Anti-Inflammatory Effects of HUPP

**Figure 11:**
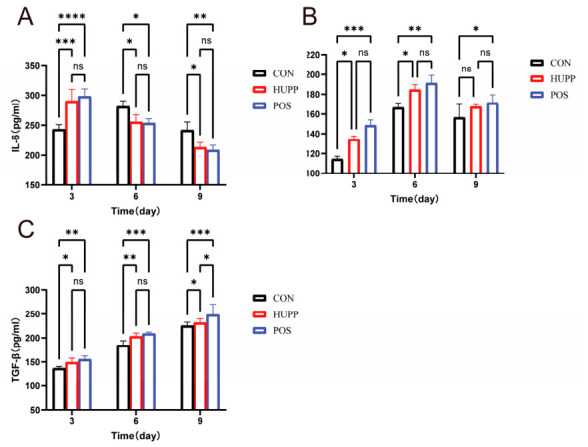
The anti-inflammatory effect of HUPP. (**A**) IL-6, (**B**) IL-10, (**C**) TGF-β (*n* = 6). Statistical significance was considered at * *p* < 0.05, ** *p* < 0.01, *** *p* < 0.001, and **** *p* < 0.0001.

Inflammatory cytokines are biomarkers associated with inflammatory responses. The cytokines IL-6, IL-10, and TGF-β measured in this experiment are key cellular inflammatory mediators that regulate wound repair. Among these, IL-6 is a pro-inflammatory cytokine that increases in the early stages of wound healing, recruiting immune cells (e.g., neutrophils) to initiate the inflammatory response [57,58]. IL-10 and TGF-β are anti-inflammatory factors. IL-10 increases during the late phase of inflammation, enhancing B lymphocyte homing, promoting the differentiation of regulatory B cells, reducing inflammation, and driving tissue repair [59,60,61]. TGF-β increases during the proliferation and remodeling phases, promoting fibroblast proliferation, collagen synthesis, and angiogenesis, making it a key factor in tissue repair [58,62,63]. As shown in Figure 11, the pro-inflammatory cytokine IL-6 in the CON group began to rise from day 3, peaked at day 6, and then declined. In contrast to the CON group, the HUPP group and the POS group showed a daily decrease starting from day 3. This discrepancy may be attributed to the treatment in the HUPP group and the POS group accelerating the onset of the inflammatory peak, thereby hastening the healing process. Levels of anti-inflammatory cytokines, including IL-10 and TGF-β, showed significant increases, indicating that HUPP possesses potent anti-inflammatory effects. These results indicate that treatment with HUPP effectively promotes wound healing, as evidenced by the earlier onset of the inflammatory peak and alterations in anti-inflammatory cytokine levels.

During acute skin wound healing, IL-6 stimulates fibroblasts to produce B-cell chemotactic factors, promoting B-cell adhesion. IL-10 tightly regulates B-cell functional polarization through negative feedback, transitioning the wound microenvironment from a pro-inflammatory to an anti-inflammatory and pro-reparative state—acting as a “safety valve” to ensure the smooth progression of healing into the proliferative phase. TGF-β synergizes with IL-10 to promote the generation and function of regulatory B cells. More importantly, TGF-β serves as a key signal for B cells to undergo antibody class switching. It enhances IgA secretion and directly stimulates fibroblasts to transform into myofibroblasts, which produce large amounts of collagen and extracellular matrix to construct granulation tissue [64]. The HUPP group exhibited an ideal healing pattern: pro-inflammatory factor IL-6 was suppressed, while anti-inflammatory factors IL-10 and TGF-β were enhanced. This confirms that HUPP exerts an immunoregulatory effect: by neutralizing pathogens via IgA and inducing B cells to secrete IL-10, it rapidly controls inflammation and promotes tissue remodeling by upregulating TGF-β.

### 3.8. HUPP Promote Cell Proliferation and Angiogenesis

To evaluate the role of HUPP in the wound healing process, we performed immunofluorescence staining for Ki-67 and CD-31 on wound tissues at days 6 and 12. Ki-67 is a nuclear protein whose expression indicates the proportion of proliferating cells within a tissue. CD-31 is a vascular endothelial cell marker commonly used to assess vascular density and formation in tissues [65,66].

**Figure 12:**
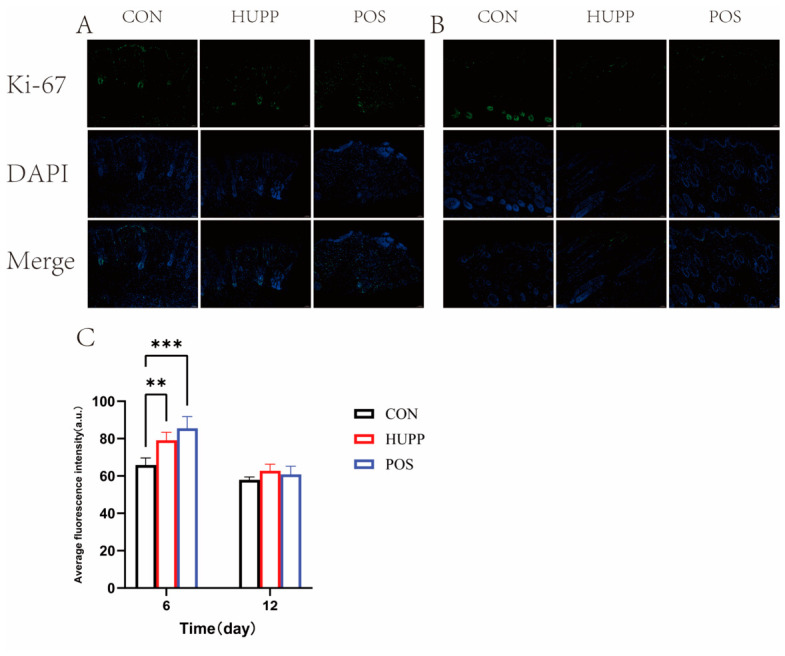
(**A**) Ki-67 immunofluorescence staining of Representative skin wound tissue. (**B**) Ki-67 sections of immunofluorescence staining (20× magnification). (**C**) Quantitative average fluorescence intensity of Ki-67 immunofluorescence staining (*n* = 6). Statistical significance was considered at * *p* < 0.05, ** *p* < 0.01, *** *p* < 0.001, and **** *p* < 0.0001.

**Figure 13:**
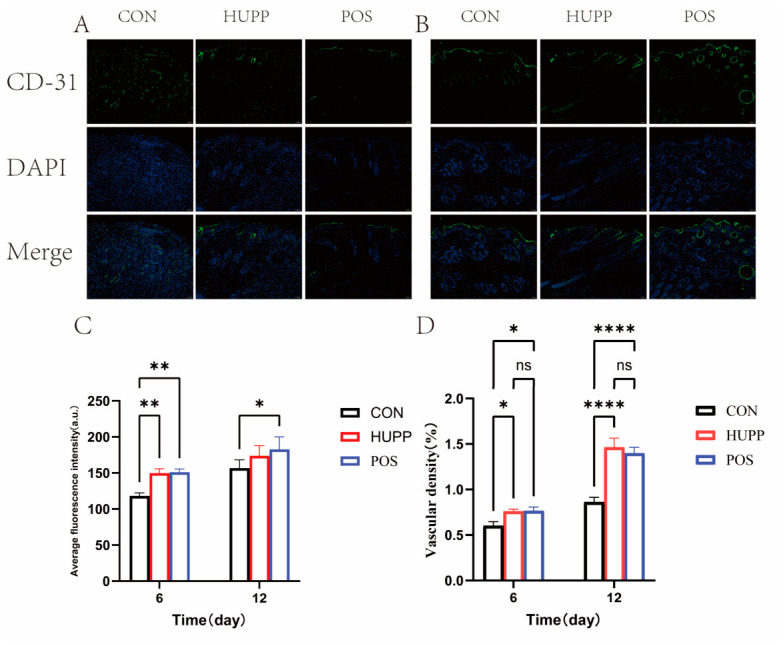
(**A**) CD-31 immunofluorescence staining of skin wound tissue. (**B**) Representative CD-31 sections of immunofluorescence staining (20× magnification). (**C**) Quantitative average fluorescence intensity of CD-31 immunofluorescence staining (*n* = 6). (**D**) The density of CD31 blood vessels was quantified from immunofluorescence images. Statistical significance was considered at * *p* < 0.05, ** *p* < 0.01, *** *p* < 0.001, and **** *p* < 0.0001.

All groups exhibited elevated Ki-67 expression on day 6 post-injury, indicating active cellular proliferation (including fibroblasts and endothelial cells involved in granulation tissue formation) at the wound site, while CD31 expression was low at this time point. By day 12, Ki-67 expression had decreased, suggesting diminished cellular proliferation activity and indicating that tissue repair may have entered the remodeling phase. It is shown in Figure 12. CD-31 expression, however, was significantly elevated, indicating that a large number of new capillaries began to form to meet the tissue’s growth demands. And it is shown in Figure 13.

Notably, on day 6 of the mid-healing phase, both Ki-67 and CD-31 expression levels in the HUPP-treated group were significantly higher than those in the CON group. These results indicate that HUPP effectively promotes cellular proliferation during the early stages of wound repair and subsequent angiogenesis, creating a favorable microenvironment for wound healing. These changes in the wound microenvironment induced by HUPP provide a potential basis for the functional regulation of immune cells, including B lymphocytes. However, whether HUPP specifically promotes healing through B-cell-mediated immune regulation requires subsequent direct validation of B-cell infiltration, subpopulations, and antibody expression.

## 4. Discussion

This study systematically elucidated the multidimensional regulatory mechanism of HUPP in skin wound repair. Its core contribution lies in establishing, for the first time, an integrative framework of “HUPP–microbiota–immune balance–tissue regeneration.” Unlike conventional pro-healing materials that primarily target a single pathway, HUPP simultaneously remodels the wound microenvironment. Preliminary toxicity evaluation demonstrated that, under the experimental conditions employed, HUPP did not cause body weight loss, abnormal organ coefficients, or histopathological changes in major organs, indicating favorable local safety. Specifically, HUPP not only significantly reduced the colonization of common wound pathogens such as *Staphylococcus* but also suppressed the overgrowth of opportunistic pathogens like *Nesterenkonia*, thereby alleviating microbially driven inflammation at its source. At the immune level, HUPP facilitated the orderly transition from the inflammatory phase to the proliferative phase, as evidenced by decreased IL-6 levels and upregulated expression of IL-10 and TGF-β, which collaboratively promoted granulation tissue formation, collagen deposition, and angiogenesis. For inflammatory wounds, which are frequently accompanied by microbial dysbiosis and persistent inflammation, HUPP exerts its ecological regulatory effect of “suppressing harmful bacteria while supporting beneficial ones.” This mechanism avoids the drug resistance or secondary microecological disturbances often associated with broad-spectrum antimicrobials, making it particularly suitable for refractory wounds that require long-term management. Moreover, the multi-target synergistic properties of HUPP enable it to shorten the inflammatory phase, reduce the risk of scar formation, and outperform traditional growth factors in terms of cost, stability, and safety. In addition, the favorable water solubility, biocompatibility, and thermal stability of HUPP facilitate its processing into various dosage forms such as dressings, sprays, or gels, and the porous sponge-like microstructure observed by SEM is conducive to absorbing wound exudate and maintaining a moist healing environment. In conclusion, this study not only confirms that HUPP is a natural pro-healing agent with bidirectional “microbiota–immune” regulatory functions, but it also reveals its application advantage over existing materials: it acts as a microenvironment “re-balancer” within a multi-target network. These findings provide both a theoretical foundation and experimental evidence for the development of next-generation wound repair materials centered on the modulation of wound microecology.

Although this study has preliminarily revealed the comprehensive effects of HUPP in a whole-animal model, the underlying mechanisms remain to be fully elucidated. For instance, which key microbial taxa or metabolites play a dominant role in the immunomodulatory action of HUPP? Does HUPP directly regulate immune cell functions through pattern recognition receptors such as Toll-like receptors or dendritic cell-associated C-type lectin-1? The route of administration in this study was limited to topical application; therefore, it remains unknown whether HUPP can exert similar effects via a systemic route, and the potential nonspecific contribution of local physical coverage to wound healing cannot be excluded. Moreover, the inherent structural complexity and batch-to-batch variability of polysaccharides, the substantial differences in microenvironmental characteristics between animal models and clinical inflammatory wounds, and the lack of systematic long-term safety and efficacy evaluations all represent critical obstacles that must be overcome before clinical translation. Addressing these questions will require future studies incorporating dose–response experiments with multiple administration modalities, along with integrated metagenomics, metabolomics, and gene-edited cell models to decipher the underlying mechanisms. In summary, this study systematically demonstrates the potential of HUPP as a multi-target, microenvironment-modulating natural pro-healing agent. It not only provides a scientific basis for the valorization of plant-derived resources but also lays a theoretical foundation for the design of “microbiota-friendly” smart dressings.

## 5. Conclusions

This study demonstrates that HUPP effectively promotes skin wound healing through a multidimensional “microbiota–immune–repair” mechanism. HUPP is an acidic heteropolysaccharide rich in Gal-UA and Rha, with low crystallinity, a porous sponge-like microstructure, and good thermal stability.

In a full-thickness skin wound mouse model, topical HUPP significantly accelerated wound closure, enhanced granulation tissue formation, and promoted collagen deposition. Mechanistically, HUPP reshaped the wound microbiota by reducing pathogenic *Staphylococcus* and opportunistic *Nesterenkonia*, alleviating microbially driven inflammation. Concurrently, HUPP modulated immune balance by suppressing pro-inflammatory IL-6 and upregulating anti-inflammatory IL-10 and TGF-β, facilitating the transition from inflammation to proliferation and remodeling. Immunofluorescence confirmed that HUPP promoted cell proliferation (Ki-67) and angiogenesis (CD-31).

Unlike conventional single-target dressings, HUPP acts as a multi-target microenvironment rebalancer with good water solubility, biocompatibility, and thermal stability. This study establishes HUPP as a natural pro-healing agent and provides a scientific basis for developing microbiota-friendly wound dressings.

## Figures and Tables

**Table 1:** Mouse numbers per group at each time point in serial sacrifice design.

Group	Day 0	Day 3	Day 6	Day 9	Day 12
CON	30	24	15	9	0
HUPP	30	24	15	9	0
POS	30	24	15	9	0

Note: At each of the indicated time points, a certain number of mice in each group were sacrificed for sampling (i.e., on day 3: 30 → 24; day 6: 24 → 15; day 9: 15 → 9; day 12: remaining 9 mice per group were all sacrificed). No unexpected death occurred.

**Table 2:** The retention time, content and mole percentage of monosaccharides in HUPP.

Name	Retention Time/Min	Concentration (μg/mg)	Molar Mass Ratio/%
Fucose (Fuc)	4.1	0	0
Rhamnose (Rha)	8.0	262.6	44.8
Arabinose (Ara)	8.4	23.1	4.3
Galactose (Gal)	10.5	58.6	9.1
Glucose (Glc)	12.0	18.8	2.9
Xylose (Xyl)	14.1	7.0	1.3
Mannose (Man)	14.7	8.8	1.4
Fructose (Fru)	17.3	0	0
Ribose (Rib)	19.3	0	0
Galacturonic acid (Gal-UA)	33.1	243.4	35.1
Glucuronic acid (Glc-UA)	33.7	8.3	1.2
Mannuronic acid (Man-UA)	35.0	0	0
Guluronic acid (Gul-UA)	36.4	0	0

**Table 3:** Molecular weight and chain dimension parameters of HUPP.

Name	Mw (kDa)	Mn (kDa)	PDI	Rg (nm)
HUPP	227.3 ± 6.36	113.2 ± 3.08	2.01 ± 0.08	52.1 ± 2.19

Note: Mw: weight-average molecular weight; Mn: number average molecular weight; PDI: polydispersity index; Rg: radius of gyration (*n* = 3).

**Table 4:** Permanova analysis was conducted to examine the differences in the composition of wound microbial communities among different treatment groups.

Characteristics	Df	SumsOfSqs	MeanSqs	F_Model	R^2^	Pr (>F)
PERMANOVA	2	0.79888	0.39944	5.9997	0.57142	0.001
Residuals	9	0.59919	0.06658	0	0.42858	0
Total	11	1.39808	0	0	1	0

Note: Based on the Bray–Curtis distance matrix, the number of permutations = 9999 times. Pr (>F) less than 0.05 indicates that there is a statistically significant difference between the groups.

## Data Availability

The data presented in this study are available on request from the corresponding author.
